# Meta-analysis of cerebrospinal fluid neuron-specific enolase levels in Alzheimer’s disease, Parkinson’s disease, dementia with Lewy bodies, and multiple system atrophy

**DOI:** 10.1186/s13195-021-00907-3

**Published:** 2021-10-05

**Authors:** Takayuki Katayama, Jun Sawada, Kae Takahashi, Osamu Yahara, Naoyuki Hasebe

**Affiliations:** 1 Department of Neurology, Asahikawa City Hospital, 1-1-65 Kinseicho, Asahikawa, 070-8610 Japan; 2 Division of Neurology, First Department of Internal Medicine, Asahikawa Medical University Hospital, Asahikawa, Japan

**Keywords:** Neuron-specific enolase, Cerebrospinal fluid, Alzheimer’s disease, Parkinson’s disease, Dementia with Lewy bodies, Multiple system atrophy

## Abstract

**Background:**

This study examined the usefulness of cerebrospinal fluid (CSF) neuron-specific enolase (NSE) levels as a candidate biomarker of neurodegeneration in Alzheimer’s disease (AD), Parkinson’s disease (PD), PD with dementia (PDD), dementia with Lewy bodies (DLB), and multiple system atrophy (MSA).

**Methods:**

We performed a systematic search of PubMed, the Cochrane Library, Scopus, and Google Scholar to find studies that measured CSF NSE levels in AD, PD, DLB, and/or MSA. For each disease, we pooled all available data and performed a meta-analysis, and meta-regression analyses of age and sex were conducted if the main analysis found a significant association.

**Results:**

Twenty studies were included (13 for AD, 8 for PD/PDD/DLB, and 4 for MSA). Significantly elevated CSF NSE levels were detected in AD (Hedges’ *g* = 0.822, 95% confidence interval [*95% CI*] 0.332 to 1.311, *p* = 0.0010), but the data exhibited high heterogeneity (*I*^2^ = 88.43%, *p* < 0.001). The meta-regression analysis of AD showed that age (*p* < 0.001), but not sex, had a significant effect on CSF NSE levels. A meta-analysis of the pooled data for PD/PDD/DLB did not show any significant changes in the CSF NSE level, but a sub-group analysis of PDD/DLB revealed significantly elevated CSF NSE levels (Hedges’ *g* = 0.507, *95% CI* 0.020 to 0.993, *p* = 0.0412). No significant changes in CSF NSE levels were detected in MSA.

**Conclusions:**

The CSF NSE level may be a useful biomarker of neurodegeneration in AD and PDD/DLB. Age was found to affect the CSF NSE levels of AD patients.

**Supplementary Information:**

The online version contains supplementary material available at 10.1186/s13195-021-00907-3.

## Introduction

Neuron-specific enolase (NSE; or γ-enolase) is a 78-kDa enzyme (phosphopyruvate hydratase), which is involved in glycolysis and is abundantly and ubiquitously expressed in neurons and neuroendocrinal cells, and 98% of NSE in cerebrospinal fluid (CSF) originates from the central nervous system [1, 2]. Therefore, NSE may be a useful biomarker of axonal injury or neuronal loss [3]. Indeed, it is widely accepted to be a useful biomarker of Creutzfeldt-Jakob disease [4], hypoxic encephalopathy [5], epilepsy [6], and brain injuries [7].

The CSF level of NSE has also been studied in neurodegenerative disorders, such as Alzheimer’s disease (AD), Parkinson’s disease (PD), Lewy body disease (LBD), dementia with Lewy bodies (DLB), and multiple system atrophy (MSA). However, these previous studies reported inconclusive or even conflicting results.

Herein, we report the first meta-analysis of the CSF levels of NSE in AD, PD, DLB, and MSA.

## Methods

We adopted the PRISMA 2009 system for the meta-analysis. We performed a search of PubMed, the Cochrane Library, Google Scholar, and Scopus for articles published on or before September 22, 2020. The keywords used for the search were as follows: “neuron-specific enolase” AND “cerebrospinal fluid” AND (“Alzheimer” OR “Parkinson” OR “Lewy” OR “multiple system atrophy”). Non-human studies, irrelevant studies, non-English articles, and review articles were excluded. All available articles were retrieved, and the mean and standard deviation (SD) values they reported were pooled. If an article reported median, quartile, or standard error values, the data were converted to mean and SD values using a previously reported method [5, 8].

Effect sizes (ES) were generated based on the sample size, mean CSF NSE level, and the associated SD values. Detailed data from our previous study [9] are also reported in this article. The significance of differences in the pooled ES was estimated using 95% confidence intervals (*95% CI*).

We combined the data for PD, PDD, LBD, and DLB and analyzed it under the heading “PD/PDD/DLB” because these conditions share common mechanisms, i.e., they are all synucleinopathies involving Lewy bodies [6], and we analyzed the data for AD and MSA separately.

All statistical analyses were performed using the STATA software, version 16 (StataCorp LLC, TX, USA), and a random effects model (the DerSimonian-Laird method) was adopted. Heterogeneity was assessed with Cochran’s *Q* test, and the Higgins *I*^2^ index was used to measure heterogeneity across studies. *I*^2^ indexes of 0–25%, 26–50%, 51–75%, and 76–100% were regarded to indicate low, medium, high, and very high levels of heterogeneity, respectively. Forest plots and funnel plots were created with the abovementioned software. Egger’s test was used to check for publication bias. Sensitivity analyses were performed by removing the data for one study at a time to test whether the outcomes of the meta-analysis were significantly influenced by a single study. When significantly altered CSF NSE levels were detected in the main analysis, meta-regression analysis was performed to assess the effects of age and sex.

*p*-values of <0.05 (*p* < 0.05) were considered statistically significant. We did not adjust the level of significance for multiple comparisons because of the exploratory aims of our analyses.

We also performed a second search of PubMed, the Cochrane Library, Google Scholar, and Scopus for articles containing information about CSF NSE levels in frontotemporal dementia (FTD) published on or before August 21, 2021. The keywords used for the search were as follows: “neuron-specific enolase” AND “cerebrospinal fluid” AND (“frontotemporal dementia” OR “frontotemporal lobar degeneration” OR “semantic dementia” OR “progressive nonfluent aphasia” OR “Pick disease”).

## Results

**Fig. 1:**
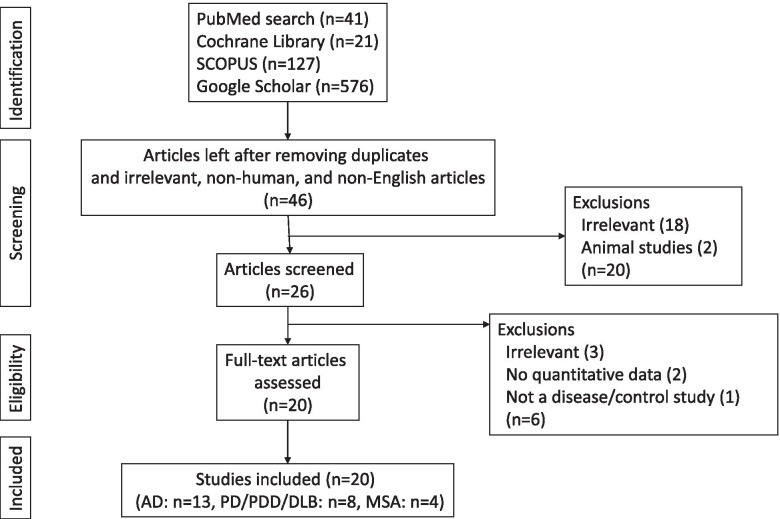
Study design. AD Alzheimer’s disease, DLB dementia with Lewy bodies, MSA multiple system atrophy, PD Parkinson’s disease. The figures in parentheses indicate the numbers of articles

We found 41 relevant articles in PubMed, 21 in the Cochrane Library, 127 in Scopus, and 576 in Google Scholar. We scrutinized their titles, abstracts, and contents and eliminated the articles that met the exclusion criteria. Finally, 20 studies (13 for AD, 8 for PD/PDD/DLB, and 4 for MSA) were included [9–28] (Additional files 1, 2, and 3). Five studies reported data for two or more diseases (Fig. 1).

The second search relating to FTD revealed 3 articles in PubMed, 0 in the Cochrane Library, 80 in Google Scholar, and 10 in Scopus. Only two studies were eligible for inclusion, as shown in Additional file 4 [15, 16], and no significant changes were reported. Therefore, we did not perform a meta-analysis of FTD.

### Analysis of AD

The pooled data for AD are summarized in Additional file 1. One study reported early- and late-onset AD separately [14], and we combined the mean and SD data for these conditions into a single group.

**Fig. 2:**
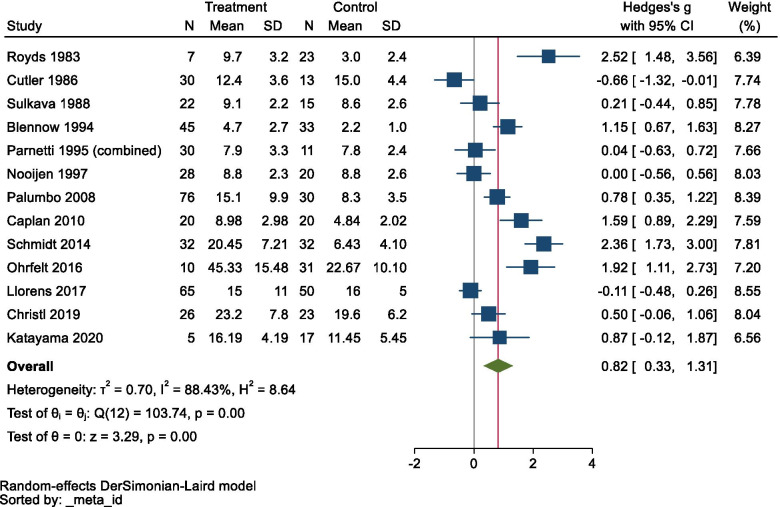
Forest plot for Alzheimer’s disease. CI confidence interval, SD standard deviation

The meta-analysis detected significantly elevated CSF NSE levels in AD (Hedges’ *g* = 0.822, *95% CI* 0.332 to 1.311, *p* = 0.0010), but the data exhibited very high heterogeneity (Cochran’s *Q* = 103.74, *df* = 12, *I*^2 =^ 88.43%, *p* < 0.001; Fig. 2).

**Fig. 3:**
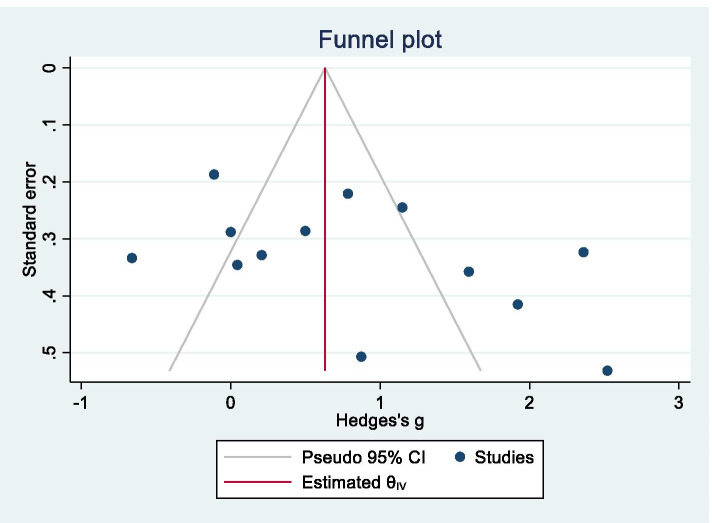
Funnel plot for Alzheimer’s disease

The sensitivity analysis, which involved the removal of the data for each study in turn, did not identify any study that significantly affected the result, and therefore, the consistency of the conclusion was confirmed. A funnel plot is shown in Fig. 3, and Egger’s test did not produce a significant result (*z* = 1.87, *p* = 0.0608), i.e., no publication bias was observed.

The meta-regression analysis showed that age (coefficient = 0.1626, standard error = 0.0407, *95% CI* 0.0828 to 0.2424, *z* = 3.99, *p* < 0.001), but not sex (*p* > 0.05), had a significant effect on the CSF NSE levels of AD patients.

When the subjects were pooled, the mean age of the AD patients was 71.0 ± 9.5 years, and that of the controls was 67.5 ± 12.2 years.

### Analysis of PD, PDD, LBD, and DLB

**Fig. 4:**
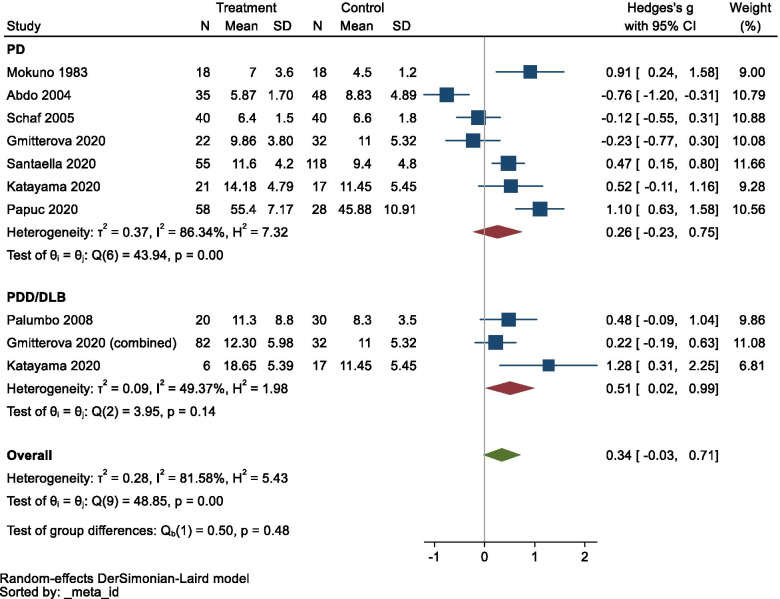
Forest plot for Parkinson’s disease (PD), PD with dementia, dementia with Lewy bodies, and the overall analysis. CI confidence interval, SD standard deviation

**Fig. 5:**
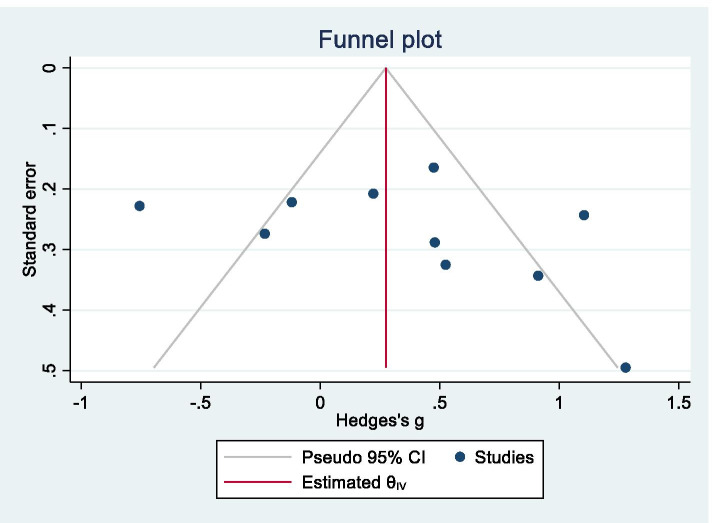
Funnel plot for Parkinson’s disease (PD), PD with dementia, and dementia with Lewy bodies

The pooled data for these diseases are summarized in Additional file 2. The meta-analysis did not show any significant changes in CSF NSE levels in PD/PDD/DLB (Hedges’ *g* = 0.343, *95% CI* −0.027 to 0.713, *p* = 0.0694), although the data exhibited very high heterogeneity (*Q* = 48.85, *df* = 7, *I*^2^ = 81.58%, *p* < 0.0001). However, a sub-group analysis showed significantly elevated CSF NSE levels in PDD/DLB (Hedges’ *g* = 0.507, *95% CI* 0.020 to 0.993, *p* = 0.0412; Fig. 4). A funnel plot is shown in Fig. 5, and Egger’s test did not produce a significant result (*z* = 1.52, *p* = 0.1280), i.e., there was no publication bias.

In the sensitivity analysis, the results were only affected when the data from the study by Abdo et al. [23] were removed (Hedges’ *g* = 0.494, *95% CI* 0.179 to 0.810). In a meta-regression analysis of PDD/DLB, neither age nor sex exhibited a significant effect on the CSF NSE level (*p* > 0.05).

When the subjects were pooled, the mean age of the PD/PDD/DLB patients was 64.0 ± 17.7 years (PD patients, 62.1 ± 13.1; PDD/DLB patients, 69.2 ± 25.7), and that of the controls was 61.1 ± 16.5 years.

### Analysis of MSA

The pooled data for MSA are summarized in Additional file 3.

Two studies examined MSA with predominant Parkinsonism (MSA-P) and MSA with cerebellar features (MSA-C) separately [23, 28]. We combined the mean and SD data for these conditions to create a single group for the main analysis.

**Fig. 6:**
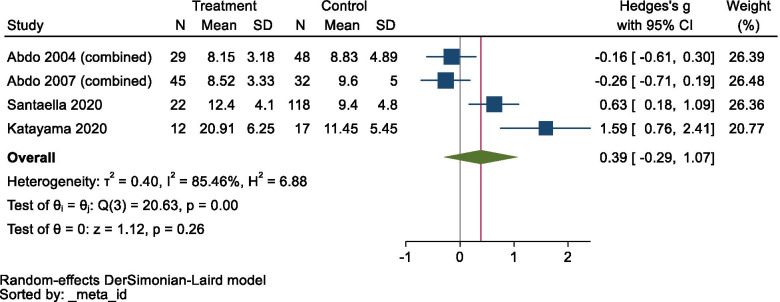
Forest plot for multiple system atrophy. CI confidence interval, SD standard deviation

Meta-analysis did not show any significant changes in CSF NSE levels in MSA (Hedges’ *g* = 0.387, *95% CI* −0.293 to 1.067, *p* = 0.2648; Fig. 6), although the data displayed very high heterogeneity (*Q* = 20.63, *df* = 3, *p* = 0.0001, *I*^2 =^ 85.46%). Furthermore, sub-group analyses of MSA-C and MSA-P without the data from the study by Santaella et al. [26] were performed because sub-group data were not reported in the latter study, but no significant changes in CSF NSE levels were detected in either group (MSA-C: Hedges’ *g* = 0.412, *95% CI* −0.654 to 1.479; MSA-P: Hedges’ *g* = −0.006, *95% CI* −0.577 to 0.566; see Additional file 5). No sensitivity analysis was performed because of the small size of the data sample.

**Fig. 7:**
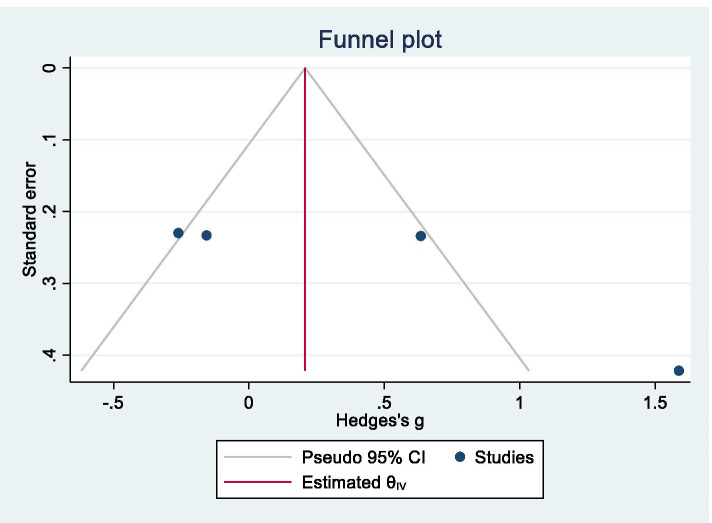
Funnel plot for multiple system atrophy

A funnel plot is shown in Fig. 7, and Egger’s test produced a statistically significant result (*z* = 3.19, *p* = 0.0014), which suggested that our data for MSA were affected by publication bias.

When the subjects were pooled, the mean age of the MSA patients was 62.1 ± 8.2 years, and that of the controls was 58.9 ± 11.9 years.

## Discussion

This is the first reported meta-analysis of the CSF NSE levels of AD, PD, DLB, and MSA patients, and it provided evidence about the significance of CSF NSE levels in AD and PDD/DLB.

### CSF NSE levels in AD

This study detected significantly elevated CSF NSE levels in AD patients, which are considered to reflect the neurodegenerative processes that occur in AD, and the sensitivity analysis of the AD-related data confirmed the consistency of the results. Therefore, this study indicated that the CSF NSE level might be useful as an objective surrogate biochemical marker of AD-related neuronal damage. Total tau (t-tau) is widely accepted as a biomarker of AD-related neurodegeneration [29], and the current study suggested that the CSF NSE level could also be used for such purposes.

Meta-regression analysis revealed that age contributed to the high heterogeneity of the CSF NSE data, and this point should be considered when interpreting the CSF NSE levels of AD patients. However, a previous study reported that CSF NSE levels were not correlated with age or sex in a normal population [30]. Other possible causes of the heterogeneity include sex, the spatial distribution of pathological changes, disease activity/rapidity, disease stage, genotypes (e.g., apolipoprotein ε4), and confounding vascular risk factors (hypertension, diabetes mellitus, dyslipidemia, etc.). Indeed, a previous study detected a significant correlation among NSE, amyloid β42, and t-tau levels [17]. Generally, NSE is seen as a marker of neurodegeneration, whereas amyloid and tau are regarded as markers of upstream changes in AD. Further studies are needed to examine these points.

The high heterogeneity observed in the CSF NSE levels of the AD patients in this study raises other possibilities, such as the effects of diagnostic accuracy. The clinical diagnosis of AD is based on the relevant criteria, but post-mortem pathological verification has demonstrated the difficulty of achieving an accurate pre-mortem diagnosis and differentiating AD from non-Alzheimer’s dementias, such as argyrophilic grain disease, primary age-related tauopathy, and other AD-mimicking disorders, pre-mortem [31]. A previous set of diagnostic criteria (NINCDS-ADRDA) exhibited high sensitivity (93%) for diagnosing AD and FTD, but low specificity (23%) [32]. However, a study of the latest criteria for AD (the IWG-2 criteria), which include criteria relating to CSF biomarkers and amyloid positron emission tomography (PET), reported that the use of this combination of biomarkers resulted in a sensitivity value of 90–95% and a specificity value of about 90% for diagnosing AD, and the agreement between florbetapir amyloid PET images and post-mortem results reached 96% [33]. Therefore, the updating of diagnostic criteria to account for new methodologies could have contributed to the heterogeneity observed in this study. Other possible reasons for the heterogeneity include variations in the disease duration, stage, or activity of AD, and differences among the subtypes of AD [34]. The meta-regression analysis of AD conducted in this study showed that age contributed to the heterogeneity in the CSF NSE levels of the AD patients. Many previous studies did not stratify their data according to disease duration or stage. In addition, another study did not detect a clear difference between the CSF NSE levels of early-onset and late-onset AD patients [14]. Thus, further studies with larger samples are needed to examine this issue. Moreover, technical factors, such as sampling procedures or assay methods, should be evaluated to clarify whether they can explain the observed heterogeneity.

Our study could not elucidate the effects of other confounding factors, such as disease severity, because relevant information was missing from the source data. For example, we checked the Mini-Mental State Examination (MMSE) scores of the subjects in previous studies (Additional file 1), but only a few studies provided such information, and therefore, we did not include MMSE as a variable in the meta-analysis. One study reported a negative correlation between MMSE scores and CSF NSE levels [14], but further studies would be needed to confirm this finding. Moreover, there have not been any longitudinal studies of CSF NSE levels, which could provide interesting results.

Our meta-analysis study did not include mild cognitive impairment because it is quite a heterogeneous condition and can include early AD, PD/DLB, vascular cognitive impairment, FTD, and depression.

### CSF NSE levels in PD, PDD, and DLB

This study revealed that CSF NSE levels were significantly elevated in PDD/DLB, but not in PD.

The detection of significant changes in CSF NSE levels in both AD and DLB reminds us that AD and DLB share common amyloid β- and tau-related pathologies [35], but examining the effects of these pathologies on CSF NSE levels would require further studies, such as studies involving amyloid or tau PET. The meta-regression analysis of PDD/DLB did not show significant effects of age or sex on CSF NSE levels, but these results were inconclusive because of the small number of studies included.

### CSF NSE levels in MSA

This study did not detect any significant changes in CSF NSE levels in MSA, although significant evidence of publication bias relating to this topic was noted. Recent studies have identified other potentially useful biomarkers of MSA, such as α-synuclein, neurofilament light chain, DJ-1, 8-hydroxyguanosine (8OHG), Fms-related tyrosine kinase ligand (Flt3L), YKL-40 (also known as chitinase-3-like protein 1; CHI3L1), and ubiquitin carboxy-terminal hydrolase L1 (UCHL-1) [36]. Further studies are needed to identify the optimal molecular biomarkers of MSA.

### CSF NSE levels in FTD

We focused on neurodegenerative disorders related to AD pathology (amyloid β and tau) and synucleinopathies in the initial stage of this study. We subsequently tried to perform a second search for studies relating to FTD; however, the number of studies found was too small to allow a meta-analysis to be performed. It should also be mentioned that the clinical, pathological, and genetic features of FTD exhibit marked heterogeneity [37]. Furthermore, the prevalence of FTD is lower than that of AD, which affects subject recruitment. Further studies of FTD are needed, and identifying other markers of the disease, such as neurofilament light chain, would also be helpful [37].

### Comparability of NSE measurements

The assays used in previous studies included enzyme-linked immunosorbent assays, electrochemiluminescence immunoassays, and radioimmunoassays. The validity of direct comparisons among these methods has not been fully elucidated, but each method is well established, and therefore, data obtained using these methods should be comparable. Some previous studies mentioned coefficients of variation (CV) for intra- or inter-assay verification and dynamic ranges for CSF NSE measurements, and other studies used commercial kits, for which CV and dynamic ranges can be obtained from the manufacturer’s information. Further studies are needed to standardize CSF NSE measurement methods.

### Limitations

This study had some limitations. First, the CSF NSE level data exhibited high heterogeneity, and there were quite large overlaps between the disease groups and controls. Therefore, the application of this biomarker to clinical practice should be performed cautiously. Second, elevated CSF NSE levels reflect neuronal damage, but are not disease-specific. Several of the studies included in this study used panels of biomarkers (amyloid β, t-tau, phosphorylated tau, α-synuclein, and neurofilament light chain, etc.) to detect combinations of molecular pathological changes.

Nevertheless, measuring CSF NSE levels could be useful because NSE assays are available in many laboratories.

## Conclusions

This meta-analysis revealed significantly elevated CSF NSE levels in AD and PDD/DLB, but not in PD without dementia or MSA. This study will aid our understanding of the pathological mechanisms underlying these diseases and support further investigations, more accurate diagnosis, and evaluations of therapeutic interventions.

## Supplementary Information

**Additional file 1..** Pooled data for Alzheimer’s disease. SD: standard deviation; SE: standard error.

**Additional file 2..** Pooled data for Parkinson’s disease (PD), PD with dementia, and dementia with Lewy bodies.

**Additional file 3..** Pooled data for multiple system atrophy.

**Additional file 4..** Pooled data for frontotemporal dementia.

**Additional file 5..** Forest plot for the sub-group analyses of multiple system atrophy (MSA). CI: confidence interval; MSA-C: MSA with cerebellar features; MSA-P: MSA with predominant Parkinsonism; SD: standard deviation.

## Data Availability

The datasets used and/or analyzed during the current study are available from the corresponding author on reasonable request.

## Abbreviations

AD: Alzheimer’s disease
CI: Confidence interval
CSF: Cerebrospinal fluid
CV: Coefficient of variation
DLB: Dementia with Lewy bodies
ES: Effect size
FTD: Frontotemporal dementia
LBD: Lewy body disease
MMSE: Mini-Mental State Examination
MSA: Multiple system atrophy
MSA-C: Multiple system atrophy with cerebellar features
MSA-P: Multiple system atrophy with predominant Parkinsonism
NSE: Neuron-specific enolase
PD: Parkinson’s disease
PDD: Parkinson’s disease with dementia
PET: positron emission tomography
SD: Standard deviation
t-tau: Total tau
