# Supplementary figures and images for: Meta-analysis of cerebrospinal fluid neuron-specific enolase levels in Alzheimer’s disease, Parkinson’s disease, dementia with Lewy bodies, and multiple system atrophy
Source: Alzheimers Res Ther. 2021 Oct 5;13:163. doi: 10.1186/s13195-021-00907-3 (PMC8493707; doi:10.1186/s13195-021-00907-3)

## Slide 1
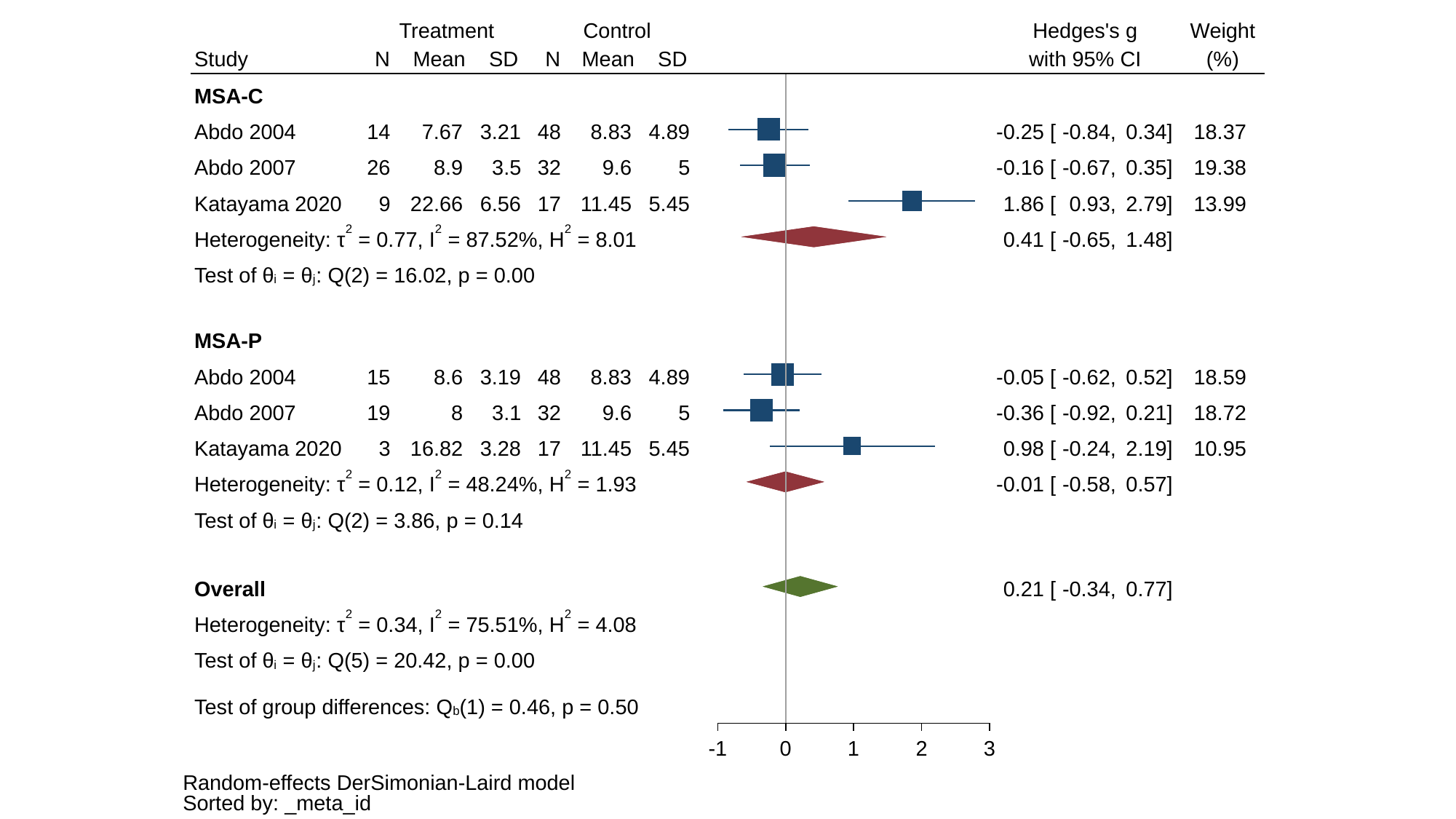

Supplement: Supplementary file 5 — Additional file 5.. Forest plot for the sub-group analyses of multiple system atrophy (MSA). CI: confidence interval; MSA-C: MSA with cerebellar features; MSA-P: MSA with predominant Parkinsonism; SD: standard deviation. [file 13195_2021_907_MOESM5_ESM.pptx]
